# Combined Oral Administration of Bovine Collagen Peptides with Calcium Citrate Inhibits Bone Loss in Ovariectomized Rats

**DOI:** 10.1371/journal.pone.0135019

**Published:** 2015-08-10

**Authors:** JunLi Liu, YiHu Wang, ShuJun Song, XiJie Wang, YaYa Qin, ShaoYan Si, YanChuan Guo

**Affiliations:** 1 Key Laboratory of Photochemical Conversion and Optoelectronic Materials, Technical Institute of Physics and Chemistry, Chinese Academy of Science, Beijing, People’s Republic of China; 2 Center for Special Medicine and Experimental Research, 306 Hospital of PLA, Beijing, People’s Republic of China; Oklahoma State University, UNITED STATES

## Abstract

**Purpose:**

Collagen peptides (CPs) and calcium citrate are commonly used as bone health supplements for treating osteoporosis. However, it remains unknown whether the combination of oral bovine CPs with calcium citrate is more effective than administration of either agent alone.

**Methods:**

Forty 12-week-old Sprague-Dawley rats were randomly divided into five groups (*n* = 8) for once-daily intragastric administration of different treatments for 3 months at 3 months after ovariectomy (OVX) as follows: sham + vehicle; OVX + vehicle; OVX + 750 mg/kg CP; OVX + CP-calcium citrate (75 mg/kg); OVX + calcium citrate (75 mg/kg). After euthanasia, the femurs were removed and analyzed by dual energy X-ray absorptiometry and micro-computed tomography, and serum samples were analyzed for bone metabolic markers.

**Results:**

OVX rats supplemented with CPs or CP-calcium citrate showed osteoprotective effects, with reductions in the OVX-induced decreases in their femoral bone mineral density. Moreover, CP-calcium citrate prevented trabecular bone loss, improved the microarchitecture of the distal femur, and significantly inhibited bone loss with increased bone volume, connectivity density, and trabecular number compared with OVX control rats. CP or CP-calcium citrate administration significantly increased serum procollagen type I N-terminal propeptide levels and reduced serum bone-specific alkaline phosphatase, osteocalcin, and C-telopeptide of type I collagen levels.

**Conclusions:**

Our data indicate that combined oral administration of bovine CPs with calcium citrate inhibits bone loss in OVX rats. The present findings suggest that combined oral administration of bovine CPs with calcium citrate is a promising alternative for reducing bone loss in osteopenic postmenopausal women.

## Introduction

Osteoporosis is the general skeletal disease that features in osteopenia. It involves degradation of the skeletal microstructure, and may cause increases in skeletal osteopsathyrosis and bone fractures. The main reasons underlying the formation of osteoporosis are reductions in bone formation and excessive increases in bone resorption [1]. As the aging society continues to increase, osteoporosis is noted at higher incidence rates among the elderly, and especially in postmenopausal women, and is referred to as a “silent epidemic disease” [2]. Treatments for osteoporosis are associated with huge expense, which conveys a burden on either society or the patient’s family. Osteoporotic patients and their families suffer from pain physically and spiritually. The effective therapeutic drugs for osteoporosis include antiresorptive drugs, such as diphosphonates, selective estrogen receptor modulators (SERMs), and receptor activator of NF-kappa-B ligand (RANKL) inhibitors [3], and drugs for improving constructive metabolism, such as parathyroid hormone [4]. However, the abovementioned drugs have certain side effects. For example, alendronate is a diphosphonate associated with the risk of increasing gastrointestinal bleeding [5,6], and raloxifene is a SERM that may cause blood coagula and hot flashes [7]. The final goal of osteoporosis therapies is to reduce the risk of osteoporotic fractures. Owing to the side effects of the abovementioned drugs and the limitations of single drugs for therapeutic effects on osteoporosis, extensive research is expected to be conducted to seek new drug targets and achieve the purposes of elevating bone mineral density (BMD), improving bone structure, and increasing bone strength [8,9].

According to clinical studies in recent years, collagen peptides (CPs) have the effects of mitigating joint symptoms and relieving arthralgia [10]. CPs are produced from collagen hydrolysates (CHs). As peptide mixtures produced by hydrolyzing collagen or gelatin and orally administered supplements with latent beneficial efficacy for improving bone joint symptoms and bone tissue regeneration, CHs have attracted wide scientific attention. From the aspect of improving bone activity, the existing reports and references show that orally administered CP supplements can increase the BMD after ovariectomy (OVX) and improve the biomechanical characteristics of vertebrae [11,12]. In our recent study, we found that the expressions of components for differentiation were improved at the mRNA and protein levels after treatment of MC3T3-E1 cells with bovine CPs, for instance RunX2, alkaline phosphatase (ALP), and osteocalcin (OC), and finally improved the formation of a mineralized bone matrix [13]. A clinical study further showed that postmenopausal osteoporotic patients taking an oral calcium-collagen chelate (CC) supplement showed higher rates of serum bone formation markers than serum bone resorption markers, and that their BMD was improved to a certain extent [14]. According to the existing clinical studies, it is not sufficient for osteoporotic patients to take calcium independently, although calcium plays important roles as an auxiliary treatment for antiresorptive agents and drugs helping to improve bone formation. However, there are no reports on whether combined oral administration of CP and calcium supplements is more effective than their individual administration. The OVX rat model is generally applied for research related to postmenopausal osteoporosis, and shows trabecular bone BMD loss and outstanding reductions in biomechanical strength after OVX [15]. In the present study, we examined the underlying effects of osteoporosis treatment with the combination of oral bovine CPs and calcium citrate, compared with administration of either agent alone, in OVX rats.

## Materials and Methods

### Ethics Statement

This study was performed in strict accordance with the recommendations in the *Guide for the Care and Use of Laboratory Animals* of Peking University Medicine College, China. The use of animals and the experimental protocols were approved by the Institutional Animal Welfare Committee of Peking University Medical College. All surgeries were performed under chloral hydrate anesthesia, and all efforts were made to minimize animal suffering.

Bovine bone CPs (Dongbao Biotechnology Co. Ltd., Baotou, China) and chemical grade calcium citrate (Dongtai Food Ingredients Co. Ltd., Lianyungang, China) were used in this study. Female Sprague-Dawley rats (age: 3 months; weight: 300 ± 20 g) were obtained from the animal facility of Peking University Medical College. The animals were housed at three rats per cage in an air-conditioned room at 23 ± 1°C and 50–60% relative humidity, with a 12-h/12-h light/dark cycle and access to a regular laboratory rodent diet. At the beginning of the experiment, group allocations were rats weight matched. After acclimatization for 3 days prior to OVX surgery, the rats were anesthetized and their bilateral ovaries were removed. A sham operation, during which the ovaries were simply touched with forceps, was performed for the sham group. Each experimental group contained eight rats. CPs were administered at 750 mg/kg, once daily, while calcium citrate was administered at 75 mg/kg, once daily.

At 3 months after surgery, 40 rats were divided into five treatment groups for intragastric administration as follows: sham + vehicle; OVX + vehicle; OVX + CP; OVX + CP-calcium citrate; OVX + calcium citrate. Bovine CPs and calcium citrate were dissolved in distilled water. All groups were treated for 3 months. At the end of the treatment period, the rats were fasted for 12 h, and blood was collected to obtain serum by centrifugation after the rats were anesthetized. The left femurs were wrapped in gauze soaked in saline solution and stored at −20°C and prepared for micro-computed tomography (μCT) analysis.

### BMD Measurements

The BMDs of the the whole left femurs were measured using a GE Lunar PIXImus (GE Healthcare, Madison, WI) and expressed as the observed mean (g/cm^2^) ± standard deviation (SD) of the whole group, based on dual energy X-ray absorptiometry (DXA) equipped with appropriate software for bone density assessments in small laboratory animals. All rats were placed in the same direction.

### μCT Analyses of the Distal Femur

For the distal femur, the whole secondary spongiosa at the left distal femur from 9-month-old sham and OVX rats were scanned by μCT using a desktop scanner (μCT40; Scanco Medical, Bruttisellen, Switzerland). with a voxel size of 10 μm, an X-ray tube voltage of 70 kVp, a current intensity of 114 μA, and an integration time of 600 ms. Briefly, slices were scanned at the region of the distal femur beginning at 0.1 mm from the most proximal aspect of the growth plate and extending proximally along the femur diaphysis. A volume of interest was manually drawn on each specimen. Structural evaluations were performed using Scanco Medical version 6.0 software. Microstructural measures included bone volume per total volume (BV/TV), connectivity density (Conn.D), trabecular number (Tb.N), and trabecular separation (Tb.Sp). The computation of these structural measures was previously described in detail elsewhere [16].

### Biochemical Analyses of Serum Samples

Blood was collected, and serum was separated by centrifugation. ALP was determined using an autoanalyzer (Model 7060; Hitachi, Tokyo, Japan). Serum bone Gla-protein (BGP) was measured by a specific ELISA for rat BGP (Rat-Mid Osteocalcin; IDS Ltd., Boldon, UK). Serum N-terminal propeptide of type I procollagen (PINP) was measured by a specific ELISA for rat PINP (Rat PINP EIA; IDS Ltd.). Beta isomer of serum C-telopeptide of type I collagen (CTX) was measured by an ELISA specific for rat CTX (RatLaps ELISA; IDS Ltd.).

### Statistical Analysis

All numerical data were expressed as means ± SD. The statistical analyses were performed using SPSS for Windows version 17.0 (IBM, Armonk, NY). One-way ANOVA followed by a least-significant difference or Dunnett’s post-hoc test was used to determine significant differences. Values of *P* < 0.05 were considered to indicate statistical significance.

## Results

### Animal Weight Changes

An *in vivo* study was performed to verify the therapeutic efficacy of CP-calcium citrate, CP alone, or calcium citrate alone using a rat osteoporosis model. Although the body weights of the OVX and sham rats did not differ significantly at the beginning of the study, the body weights of the OVX rats were significantly increased at 12 weeks after surgery compared with the sham group (*P* < 0.05). The body weights in the OVX group continued to be significantly higher than those in the sham group throughout the study, with no significant differences between the OVX groups (Table in S1 Table).

### BMD Assessment

In osteoporosis, it is considered that the risk of bone fractures can be predicted by measuring the BMDs of the lumbar vertebrae and femur. The changes in femoral BMD determined by DXA analysis in the present study are shown in Table 1. In the OVX group, there was a significant reduction by 19.61% in the femoral BMD compared with the sham group (*P* < 0.01). Oral administration of CP-calcium citrate, CP, and calcium citrate significantly improved the femoral BMDs compared with the OVX control group(14.63%, 10.37%, 8.54%, respectively, *P* < 0.05). Furthermore, there were significant differences in the femoral BMDs between the rats treated with CP-calcium citrate, CP, and calcium citrate).

**Table 1:** Analysis of femoral bone mineral density.

Group	Femur BMD (g/cm^2^)
**Sham**	**0.204±0.007** **
**OVX**	**0.164±0.004**
**CP**	**0.181±0.002** **
**CP-calcium citrate**	**0.188±0.010** **
**Calcium citrate**	**0.175±0.009** *

Sham: sham-operated group; OVX: ovariectomized control group. OVX rats were administered once-daily with 750 mg/kg CP combined with 75 mg/kg calcium citrate, or each agent alone, where indicated. Data are expressed as means ± SD (*n* = 8).

*P < 0.05 vs. OVX control group.

**P < 0.01 vs. OVX control group.

### Three-Dimensional μCT

**Fig 1:**
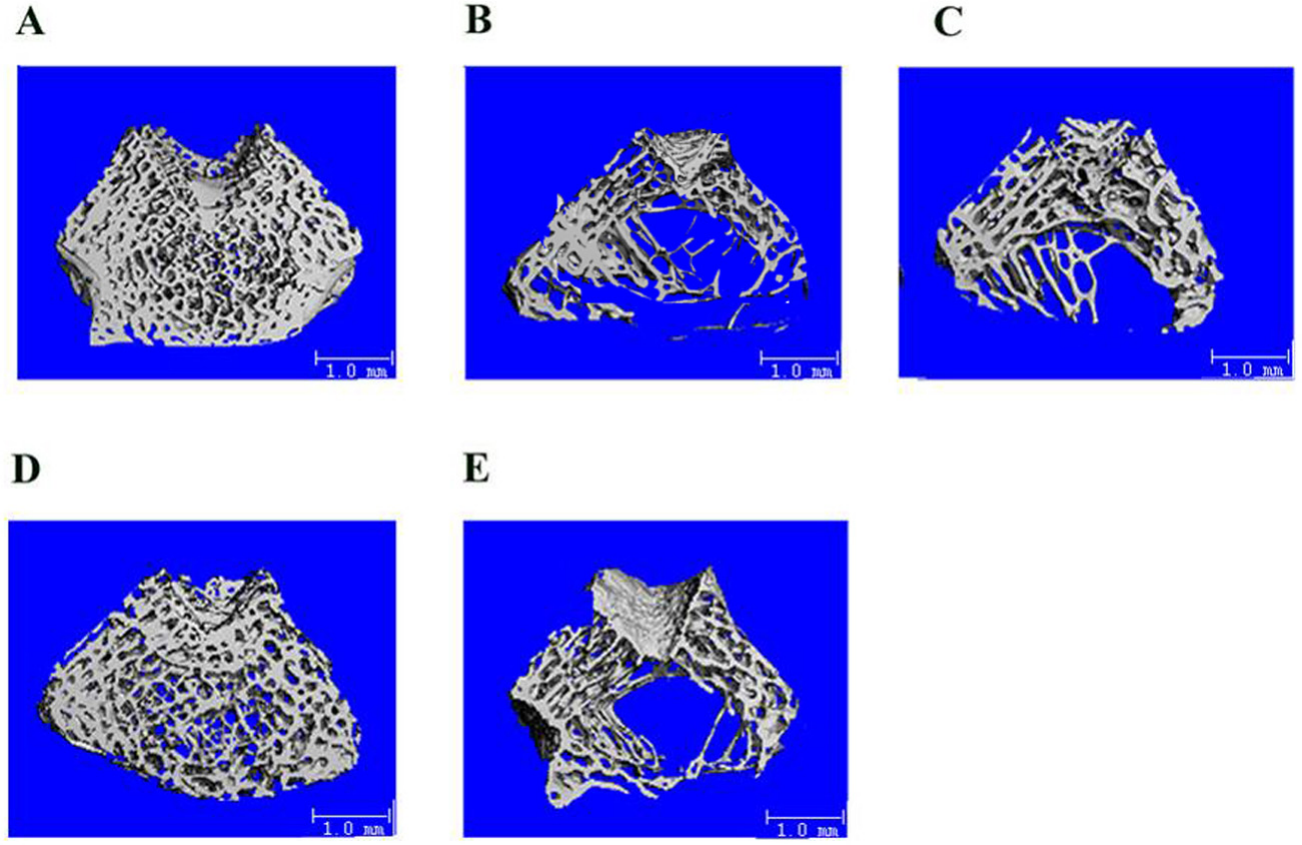
Representative three-dimensional μCT images of the distal femurs in each treatment group (*n* = 8). A: Sham-operated group. B: OVX control group. C: OVX rats administered once-daily with 750 mg/kg CP. D: OVX rats administered once-daily with 750 mg/kg CP and 75 mg/kg calcium citrate. E: OVX rats administered once-daily with 75 mg/kg calcium citrate. Scale bars = 1 mm.

Three-dimensional μCT images of the distal femurs in each treatment group are shown in Fig 1. The images revealed that the OVX rats had significantly decreased trabecular bone areas and bone density, while the sham rats showed dense intact trabecular bone. Oral administration of CP-calcium citrate significantly improved the bone microarchitecture and density, with thicker trabecular bone and increased integrity, indicating positive effects in the treatment of bone loss. The bone loss induced by OVX was also noticeably inhibited by CP and calcium citrate treatments, which exhibited thicker and more intact trabecular bone.

Analyses of microstructural indices for the distal femurs revealed that the OVX rats had significantly lower measurements (29.23%, 83.48%, 71.8%, respectively, Table 2) for BV/TV, Conn.D, and Tb.N, compared with the sham rats. CP-calcium citrate treatment significantly inhibited the reductions in these measurements (15.22%, 226.22%, 120.57%, respectively) in the OVX rats. However, there were no significant differences between the CP- and calcium citrate-treated rats. In addition, CP-calcium citrate treatment significantly decreased by 59.14% Tb.Sp in the OVX rats, which was higher than that in the sham rats.

**Table 2:** Analysis of microstructural indices of the distal femur.

	Sham	OVX	CP	CP-calcium citrate	Calcium citrate
**BV/TV (%)**	**0.65±0.03** **	**0.46±0.02**	**0.50±0.01**	**0.53±0.02** **	**0.48±0.03**
**Conn.D [1/mm** ^**3**^ **]**	**86.82±15.06** **	**14.34±6.77**	**27.79±4.83**	**46.78±8.99** **	**25.25±6.72**
**Tb.N (l/mm)**	**5.00±0.20** **	**1.41±0.36**	**2.10±0.05**	**3.11±0.79** **	**1.68±0.28**
**Tb.Sp (mm)**	**0.15±0.02** **	**0.93±0.26**	**0.59±0.03** *	**0.38±0.16** **	**0.78±0.21**

Sham: sham-operated group; OVX: ovariectomized control group. OVX rats were administered once-daily with 750 mg/kg CP combined with 75 mg/kg calcium citrate, or each agent alone, where indicated. BV/TV: bone volume/tissue volume; Conn.D: connectivity density; Tb.N: trabecular number; Tb.Sp: trabecular separation. Data are expressed as means ± SD (*n* = 5).

*P < 0.05 vs. OVX control group.

**P < 0.01 vs. OVX control group.

### Serum Levels of Bone Turnover Biomarkers

The serum levels of bone turnover biomarkers, including ALP, PINP, OC, and CTX, were measured as indicators of the protective effects of CPs on osteoporosis in the OVX rats (Table 3). OVX produced significant increases in bone remodeling, both in terms of resorption (CTX) and formation (ALP, OC, and PINP). The ALP, OC, and CTX levels were significantly decreased with CP-calcium citrate and CP treatments. Most importantly, the PINP levels in the CP-calcium citrate- and CP-treated rats were significantly higher than those in the OVX rats.

**Table 3:** Serum levels of bone turnover markers.

	Sham	OVX	CP	CP-calcium	Calcium
**ALP (U/L)**	**31.80±07.79** *	**69.80±15.30**	61.33±15.63*	**51.33±5.96** *	**65.14±7.10**
**OC (ng/ml)**	**1.61±0.17** **	**2.65±0.13**	**1.96±0.06** **	**2.07±0.20** **	**2.02±0.23** **
**PINP (ng/ml)**	**5.80±0.45** *	**9.00±1.73**	**14.00±1.87** *	**14.00±2.83** *	**11.40±2.07**
**CTX (ng/ml)**	**18.00±2.45** **	**23.75±1.71**	**14.40±2.07** **	**15.34±2.55** **	**20.06±3.29** *

Sham: sham-operated group; OVX: ovariectomized control group. OVX rats were administered once-daily with 750 mg/kg CP combined with 75 mg/kg calcium citrate, or each agent alone, where indicated. ALP: bone-specific alkaline phosphatase; OC: osteocalcin; PINP: N-terminal propeptide of type I procollagen; CTX: C-telopeptide of type I collagen. Data are expressed as means ± SD (*n* = 8).

*P < 0.05 vs. OVX control group.

**P < 0.01 vs. OVX control group.

## Discussion

The present study has demonstrated for the first time that the combination of orally administered bovine CPs (relative molecular weight: 0.6–2.5 kDa) and calcium citrate for 12 weeks significantly improved the osteoprotective effects of either agent alone in OVX rats.

It is necessary to screen suitable dosages of CPs and calcium citrate for two main reasons: first, to produce protective effects against bone loss in osteoporotic patients; and second, to prevent a heavy economic burden on patients with long-term usage. Based on these concepts, we designed the dosage of 750 mg/kg for bovine CPs alone or in combination with 75 mg/kg calcium citrate. These dosages were chosen because the commercial recommendation for CPs (10 g once daily in adults) corresponds to CPs at approximately 500 mg/kg weight in rats [1,17], while calcium citrate at 75 mg/kg weight in rats corresponds to calcium supplements at approximately 750 mg once daily in postmenopausal osteoporotic women. The bone loss in the OVX and sham rats was evaluated based on the BMD, as assessed by DXA, three-dimensional μCT, and serum levels of bone turnover biomarkers.

Clinically, methods to diagnose osteoporosis, predict the risk of bone fractures, and observe changes in bone mass by bone mass assessments are attracting increasing attention. Clinical applications of bone mass assessments are mainly split into bone densitometry and bone histomorphometry. In the past, patients without bone fractures were assessed for their risk of bone fractures using the indices of their femoral or vertebral BMDs [18]. In our research and due to limitations of the experimental conditions, only the femoral BMD was selected for bone mass assessments. The results showed that 3 months of oral therapy with bovine CPs alone or with calcium citrate, or even calcium citrate alone, could apparently suppress the loss of femoral BMD in OVX rats, and that the rats administered bovine CPs and calcium citrate together exhibited higher and more apparent efficacy than the rats administered bovine CPs or calcium citrate alone. Similar conclusions were previously verified in OVX mice [19,20]. Therapy in OVX rats using low-dose CPs was verified to provide similar facilitation for the whole-body bone density [21], indicating that CPs have a certain efficacy of treating osteoporosis.

Fractures of the femoral neck are the most common damage sustained by postmenopausal women. The main reason underlying this type of bone fracture is the rapid reduction in postmenopausal estrogen, which further causes loss of bone mass, especially in cancellous bone. The consequences of this loss are fractures of the femurs or vertebrae, and many patients cannot look after themselves in their daily life, and even suffer from enormous pain [22]. However, based on many reports [23,24,25], we found that the bone trabeculae in the distal femur of OVX rats were generally used for μCT analyses, and showed similar changes to the neck of the femur.

Our present data showed a diminished trabecular structure at the distal femur, with significant bone loss revealed by μCT, in OVX rats after 12 weeks of treatment. CP-calcium citrate treatment effectively counteracted the bone loss induced by OVX and preserved the bone microarchitecture (Figure in S1 Fig and method in S1 File). These findings indicate that administration of CPs plus calcium citrate had a greater effect in restoring the deteriorated trabecular architecture and femoral morphology than administration of CP or calcium citrate alone.

The bone trabeculae at the neck of the femur in postmenopausal senile women change hugely, including reductions in BV/TV, trabecular Conn.D, and Tb.N and increases in Tb.Sp [2,26], and the integrity of the bone trabeculae is further reduced by removal of the ovaries. In addition, the Conn.D number of bone trabeculae is the basic characteristic of the three-dimensional network maintaining skeleton strength, and is apparently reduced with increases in aging. We observed that the changes in the trabecular bone of the distal femur following OVX in rats were consistent with those in the femoral neck of older subjects [27]. CP-calcium citrate treatment significantly reduced the decreases in these values in OVX rats. In our study, as expected, Conn.D in the distal femur exhibited a similar decrease following OVX. CP-calcium citrate treatment effectively inhibited the Conn.D reduction induced by OVX, possibly through inhibition of the loss of small interconnecting trabeculae, whereas calcium citrate alone could not achieve similar results. Concurrently, we found that the trabecular structure at the distal femur of CP-treated rats showed inhibition of the changes in bone trabeculae caused by OVX to some extent, albeit not as markedly, which was different from a previous report [21]. According to our speculation, the difference was possibly related to the CP source and the inspection position on the one hand, and even to large individual differences in the experimental animals and the small quantity of specimens on the other hand. We are expecting to increase the number of specimens in future studies and to make further verifications using a tail-suspended animal model.

Markers of bone metabolism are one means for assessing bone mass and predicting the rate of bone fractures, and include both bone formation and bone resorption markers. Bone formation markers are direct or indirect expression products of osteoblasts at different developmental stages, and reflect osteoblast functions and bone formation conditions, such as serum ALP, OC, and PINP levels, while CTX is the C-telopeptide degradation product released by type I collagen in the bone resorption process of osteoclasts, and is the main bone resorption marker [28,29]. Postmenopausal osteoporosis and bone loss caused by OVX are accompanied by apparent increases in bone remodeling, and can be proven by enhanced levels of bone turnover markers [30]. Our results revealed significant decreases in femoral BMD after OVX that resulted from increased bone turnover, as indicated by the higher levels of serum ALP, OC, PINP, and CTX, compared with the sham group. Oral administration of CP-calcium citrate or either agent alone caused reductions in the serum levels of ALP, OC, and CTX, indicating a reduction in bone turnover, and a significant increase in serum PINP, indicating that CPs can promote collagen synthesis, which differs from a previous report [21]. Conversely, the significantly decreased serum CTX levels following CP-calcium citrate or CP treatment indicate that CP supplementation can inhibit the degradation of collagen, thereby further inhibiting bone resorption, as shown by the increased BMD.

According to the latest research, dietary intake of a low-molecular-weight hydrolyzed CC supplement can prevent bone loss by improving the organic content of bone. On the contrary, taking calcium or vitamin D alone can realize the opposite effect [31,32], as can taking common bone-protective drugs and calcium carbonate supplements [33,34]. However, taking into consideration the natural effects of bovine CPs and calcium citrate, and the limited understanding of their effects, we will continue to investigate the intriguing mechanisms underlying the combined effects of CP and calcium citrate in eliciting osteoprotective effects at the molecular and cellular levels in the future.

In conclusion, our results demonstrate that the significantly lower femoral BMD, femoral trabecular bone area, and microstructural indices (BV/TV, Conn.D, Tb.N) induced by OVX are accompanied by enhanced levels of serum bone turnover markers. As potential supplements for the inhibition of bone loss, oral administration of CP-calcium citrate noticeably improved these values by decreasing the levels of serum bone turnover markers. The significant increase in serum PINP and significant decrease in serum CTX, associated with treatment with CP-calcium citrate or CP alone indicate that CPs can promote collagen synthesis and inhibit collagen degradation. The present study suggests that combined oral administration of bovine CPs with calcium citrate is a promising alternative for reducing bone loss in osteopenic postmenopausal women.

## Supporting Information

S1 Fig Representative Masson’s trichrome staining images of the distal femurs. (TIF)

S1 Table Rat body weights records. (XLSM)

S1 File Legend of representative Masson’s trichrome staining images of the distal femurs. (DOC)
