# Supplementary figures and images for: Combined Oral Administration of Bovine Collagen Peptides with Calcium Citrate Inhibits Bone Loss in Ovariectomized Rats
Source: PLoS One. 2015 Aug 10;10(8):e0135019. doi: 10.1371/journal.pone.0135019 (PMC4530891; doi:10.1371/journal.pone.0135019)

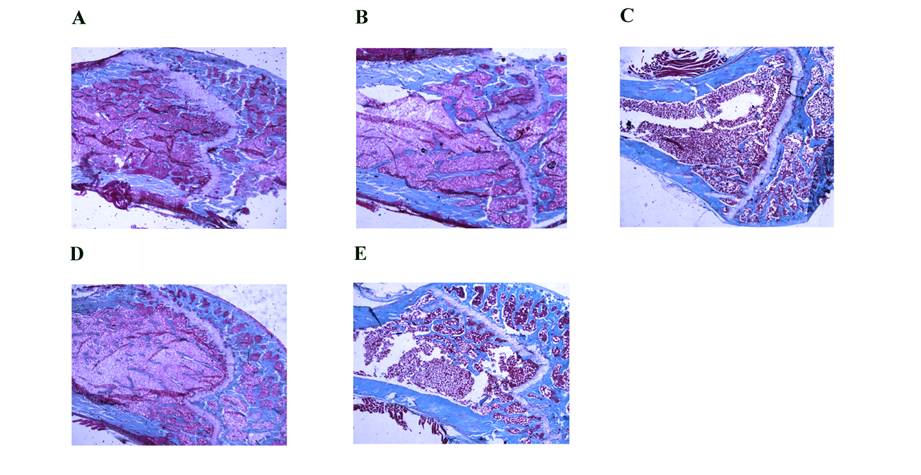

Supplement: S1 Fig — (TIF) [file pone.0135019.s001.tif]
