# Supplementary material for: Combined Oral Administration of Bovine Collagen Peptides with Calcium Citrate Inhibits Bone Loss in Ovariectomized Rats
Source: PLoS One. 2015 Aug 10;10(8):e0135019. doi: 10.1371/journal.pone.0135019 (PMC4530891; doi:10.1371/journal.pone.0135019)
Supplement: S1 File — (DOC) [file pone.0135019.s003.doc]

**Fig.2** Representative Masson’s trichrome staining images of the distal femurs in each treatment group, A: sham operated group; B: OVX control group; C: OVX rats administrated daily with 250 mg/kg CPs; D: OVX rats administrated daily with 750 mg CPs and 75 mg/kg calcium citrate; E: OVX rats administrated daily with 75 mg/kg calcium citrate. Blue: fibrous collagen-rich tissue blue; red: skeletal muscle.

**Method: histological examination**

Femoral bone specimens were fixed in 4% paraformaldehyde (pH 7.26) for 7 days, decalcified in 10% EDTA from 14 to 30 days, and then embedded in paraffin. Serial 5-µm thick sections were made and stained using masson trichrome, which stains fibrous collagen-rich tissue blue and skeletal muscle red. Images were captured using a QImaging digital camera at 20×, and the relative fraction of red and blue pixels in each image was were captured using a digital camera (Imaging, Surrey, BC Canada)
